# Schuurs–Hoeijmakers Syndrome (*PACS1* Neurodevelopmental Disorder): Seven Novel Patients and a Review

**DOI:** 10.3390/genes12050738

**Published:** 2021-05-13

**Authors:** Jair Tenorio-Castaño, Beatriz Morte, Julián Nevado, Víctor Martinez-Glez, Fernando Santos-Simarro, Sixto García-Miñaúr, María Palomares-Bralo, Marta Pacio-Míguez, Beatriz Gómez, Pedro Arias, Alba Alcochea, Juan Carrión, Patricia Arias, Berta Almoguera, Fermina López-Grondona, Isabel Lorda-Sanchez, Enrique Galán-Gómez, Irene Valenzuela, María Pilar Méndez Perez, Ivón Cuscó, Francisco Barros, Juan Pié, Sergio Ramos, Feliciano J. Ramos, Alma Kuechler, Eduardo Tizzano, Carmen Ayuso, Frank J. Kaiser, Luis A. Pérez-Jurado, Ángel Carracedo, The ENoD-CIBERER Consortium, The SIDE Consortium, Pablo Lapunzina

**Affiliations:** 1 CIBERER, Centro de Investigación Biomédica en Red de Enfermedades Raras, ISCIII, Melchor Fernández Almagro 3, 28029 Madrid, Spain; jaira.tenorio@salud.madrid.org (J.T.-C.); bmorte@ciberer.es (B.M.); jnevadobl@gmail.com (J.N.); v.martz.glez@gmail.com (V.M.-G.); fernando.santos@salud.madrid.org (F.S.-S.); sixto.garciamin@gmail.com (S.G.-M.); mpalomares.ingemm@gmail.com (M.P.-B.); martapaciomiguez@gmail.com (M.P.-M.); bgomez@ciberer.es (B.G.); palajara@gmail.com (P.A.); balmoguera@hotmail.com (B.A.); ilorda@fjd.es (I.L.-S.); francisco.barros@usc.es (F.B.); juanpie@unizar.es (J.P.); ramossorigue@gmail.com (S.R.); framos@unizar.es (F.J.R.); CAyuso@fjd.es (C.A.); luis.perez@upf.edu (L.A.P.-J.); angel.carracedo@usc.es (A.C.); 2 Overgrowth Syndromes Laboratory, INGEMM, Instituto de Genética Médica y Molecular, IdiPAZ, Hospital Universitario la Paz, Universidad Autónoma de Madrid (UAM), 28046 Madrid, Spain; 3 The SIDE Consortium: Spanish Intellectual Disability Exome Consortium, 28046 Madrid, Spain; fermina.lopez@quironsalud.es; 4 Ithaca, European Reference Network, Hospital Universitario La Paz, 28046 Madrid, Spain; mvalenzuela@vhebron.net (I.V.); etizzano@vhebron.net (E.T.); 5 Genetics Unit, Universitat Pompeu Fabra, Barcelona, Spain and Institut Hospital del Mar D’Investigacions Mediques (IMIM), 08002 Barcelona, Spain; 6 Structural and Functional Genomics—INGEMM, Instituto de Genética Médica y Molecular, IdiPAZ, Hospital Universitario la Paz, Universidad Autónoma de Madrid (UAM), 28046 Madrid, Spain; 7 Clinical Genetics—INGEMM, Instituto de Genética Médica y Molecular, IdiPAZ, Hospital Universitario la Paz, Universidad Autónoma de Madrid (UAM), 28046 Madrid, Spain; 8 FEDER (Spanish Federation for Rare Diseases), Calle del Dr. Castelo 49, 28009 Madrid, Spain; direccion@enfermedades-raras.org (A.A.); feder@enfermedades-raras.org (J.C.); infofundacionfeder@enfermedades-raras.org (P.A.); 9 Department of Genetics & Genomics, Instituto de Investigación Sanitaria-Fundación Jiménez Díaz University Hospital, Universidad Autónoma de Madrid (IIS-FJD, UAM), 28046 Madrid, Spain; 10 Clinical Genetics, Head of the Pediatrics Service, Hospital Materno Infantil de Badajoz, Complejo Hospitalario Universitario de Badajoz, Professor of Pediatrics, Director of the Department of Biomedical Sciences, Faculty of Medicine, University of Extremadura, 06110 Plasencia, Spain; enrique.galangomez@gmail.com; 11 Department of Clinical and Molecular Genetics, Vall d’Hebron University Hospital and Medicine Genetics Group, Vall d’Hebron Research Institute, 08002 Barcelona, Spain; icusco@vhebron.net; 12 Clinical Genetics, Hospital Materno Infantil de Badajoz, Complejo Hospitalario Universitario de Badajoz, University of Extremadura, 06006 Badajoz, Spain; mariapilar.mendez@salud-juntaex.es; 13 Fundación Pública Galega de Medicina Xenómica, SERGAS, Instituto de Investigación Sanitaria de Santiago (IDIS), 15702 Santiago de Compostela, Spain; 14 Unit of Clinical Genetics, Service of Paediatrics, University Clinic Hospital’ Lozano Blesa’ and Unit of Clinical Genetics and Functional Genomics, Department of Pharmacology-Physiology, School of Medicine, University of Zaragoza, CIBERER-GCV02 and ISS-Aragón, 50001 Zaragoza, Spain; 15 Institute of Human Genetics, University Hospital Essen, University Duisburg-Essen, 45147 Essen, Germany; almu@mti.uni-jena (A.K.); Frank.Kaiser@uk-essen.de (F.J.K.); 16 Center for Rare Disease/Zentrum für Seltene Erkrankungen (EZSE), University Hospital Essen, 45276 Essen, Germany; 17 Centro de Investigación en Medicina Molécula y Enfermedades Crónicas (CIMUS), Universidade de Santiago de Compostela, 15702 Santiago de Compostela, Spain

**Keywords:** Schuurs–Hoeijmakers syndrome, intellectual disability, *PACS1*, rare disorders, phosphofurin acidic cluster sorting protein 1, pathogenic variant c.607C > T

## Abstract

Schuurs–Hoeijmakers syndrome (SHMS) or *PACS1* Neurodevelopmental disorder is a rare disorder characterized by intellectual disability, abnormal craniofacial features and congenital malformations. SHMS is an autosomal dominant hereditary disease caused by pathogenic variants in the *PACS1* gene. PACS1 is a trans-Golgi-membrane traffic regulator that directs protein cargo and several viral envelope proteins. It is upregulated during human embryonic brain development and has low expression after birth. So far, only 54 patients with SHMS have been reported. In this work, we report on seven new identified SHMS individuals with the classical c.607C > T: p.Arg206Trp *PACS1* pathogenic variant and review clinical and molecular aspects of all the patients reported in the literature, providing a summary of clinical findings grouped as very frequent (≥75% of patients), frequent (50–74%), infrequent (26–49%) and rare (less than ≤25%).

## 1. Introduction

Schuurs–Hoeijmakers syndrome or *PACS1* Neurodevelopmental disorder (MIM# 615009) is a rare autosomal dominant disease characterized by distinctive craniofacial features, intellectual disability (ID) with variable degrees of neurodevelopmental delay and congenital anomalies. It was initially reported in 2012 in two unrelated patients with remarkably similar facial features and ID [1]. A few years later a review of 17 additional individuals plus the two original patients were described in a systematic review [2]. As common features included a distinctive facial appearance, delayed speech and delayed psychomotor development/ID ranging from mild to moderate. Most patients had anomalies in the eyes, nose, heart, and gastrointestinal system. After this first review, further patients were reported mainly from series of patients with ID evaluated through massive paralleled sequencing studies [3,4,5,6,7,8,9,10,11,12,13,14]. Remarkably, almost all patients show the same heterozygous de novo *PACS1* variant c.607C > T that results in exchange of an arginine residue to a tryptophan at position 203 and is assumed to be a gain of function variant. Up to date, about 54 patients have been reported, all of them with a striking similar facial phenotype and with pathogenic change in *PACS1*.

*PACS1* codes for the Phosphofurin Acidic Cluster Sorting Protein 1, which is involved in the localization of trans-Golgi network membrane proteins. In vivo functional assays were performed in the initial report of the SHMS, demonstrated that zebrafish embryos expressing the Arg203Trp change showed craniofacial abnormalities. This might be due to the inhibition of *Pacs1’*s ability to mediate the migration and specification of Sox10 in neural crest cells [1].

In this work, we report on seven novel SHMS patients all with the typical Arg203Trp variant. We systematically review all the cases reported so far providing a summary of clinical finding grouped in very frequent (≥75% of patients), frequent (50–74%), infrequent (26–49%) and rare (less than ≤25%).

## 2. Materials and Methods

**Figure 1:**
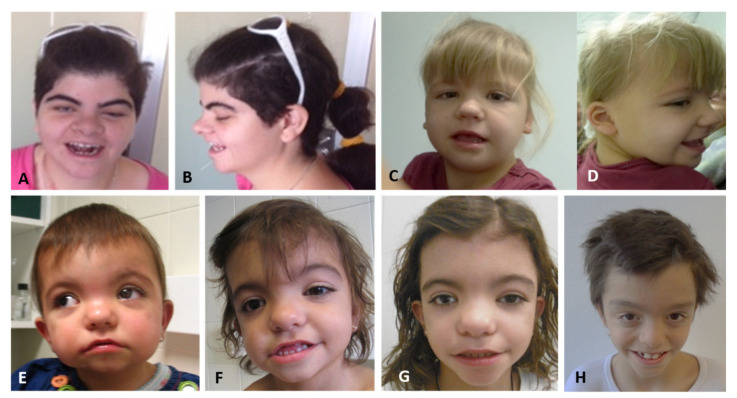
Facial phenotypes of four of the novel patients with *PACS1* pathogenic variants. (**A**,**B**) patient 1; (**C**,**D**) patient 7; (**E**–**G**) patient 4 and (**I**) patient 6.

All new patients reported herein were evaluated by clinical geneticists because of the association of ID and distinctive craniofacial features. Patient 4 was recruited by the Undiagnosed Rare Disease Program (ENoD) of CIBERER. This study was approved by the Medical Ethics Committee of the Hospital Universitario La Paz, IdiPAZ (CEIC-HULP-PI3509), and all participants signed a specific informed consent. None of the parents were consanguineous and none of the patients had any remarkable information regarding the perinatal period. A detailed description of the clinical features of all the patients reported here is listed in Supplementary Table S1 and facial phenotypes are showed in Figure 1.

On molecular level, patients were diagnosed by either clinical exome sequencing (CES; Sophia Genetics, Boston, MA) or whole exome sequencing (WES) in singleton or trio analyses. Briefly, sequencing was carried out in NextSeq500 or HiSeq4000 platforms (Illumina, San Francisco, California, USA) following the manufacturer’s instructions. In-house pipelines for bioinformatic analysis were developed to perform quality control (QC) and variant annotation. A suite of QC, scripts that facilitate data quality assessment, was applied. QC also included an assessment of total reads, library complexity, capture efficiency, coverage distribution (95% at ≥20x, capture uniformity, raw error rates, Ti/Tv ratio in coding regions (typically 3.2 for known sites and 2.9 for novel sites), distribution of known and novel variants relative to dbSNP and zygosity. An automated pipeline for the annotation of variants was developed as well. Our application returned many types of variant annotations, including dbSNPrs identification, gene names and accession numbers, predicted functional effect, protein positions and, for amino-acid changes (dbNSFP, CADD), conservation scores and several population frequency databases, and known clinical associations along with a vast array of annotations for non-coding sequences derived from ENCODE. Databases for pathogenic variants such as ClinVar (https://www.ncbi.nlm.nih.gov/clinvar/, accessed on February 2021), Human Gene Mutation Database (HGMD) (http://www.hgmd.cf.ac.uk/ac/index.php, accessed on February 2021), Leiden Open Variant Database (LOVD) (https://www.lovd.nl/, accessed on February 2021), Alamut (https://www.interactive-biosoftware.com/alamut-visual/), and Varsome (https://varsome.com/) were also reviewed. Finally, review, classification, and interpretation of the variant was made according to the American College of Medical Genetics and Genomics (ACMGG) guidelines [15]. CNV analysis was performed applying a custom script named “LACONv” (https://github.com/kibanez/LACONv), which was developed in-house.

## 3. Results

We detected seven patients with the same pathogenic variant in *PACS1.* All of them carried the most common, recurrent, likely gain of function pathogenic variant [(NM_018026.4):c.607C>T (p.Arg203Trp), chr11 (GRCh37): 65978677)].

The c.607C>T change has been clearly demonstrated to be pathogenic [2]. Segregation analyses showed that none of the parents tested was carrier of the variant, meaning that all probands had a de novo pathogenic variant.

All patients had ID, abnormal speech and distinctive craniofacial features (Figure 1), including downslanting palpebral fissures, bulbous nose, upturned nose, broad nasal bridge, ocular hypertelorism, thin upper lip, low-set ears, wide, tented mouth, full eyebrows and long eyelashes, among others (Table 1). We listed all clinical features of our patients in Supplementary Table S1 and reviewed the clinical findings observed in our patients and in all previously reported patients (N = 61) in Table 1. This table contains a review of the clinical features described in all the 61 cases described in the literature with SHMS (37 males and 24 females). The most common findings, observed in more than 75% of patients, are ID, speech delay and distinctive craniofacial features. Eye anomalies are observed in 25–75% of the patients and are useful for diagnosis.

Features observed in single patients: persistent left superior vena cava, dysplastic aortic and pulmonary valves, large ears,, hearing impairment, ectropion, lens subluxation, Peters’ anomaly, irregular optic discs, oval pupils, delayed visual maturation, lacrimal duct stenosis, rethrognatia, *frenulum linguae*, absent nasal bone, high arched palate, narrow palate, bifid uvula, misplaced teeth, small teeth, micropenis, septate uterus, congenital diaphragmatic hernia, single umbilical artery, volvulus, short bowel syndrome, ectopic anus, poor feeding, neutropenia, leukopenia, finger and toe syndactyly, hip dysplasia, short toe, fibular subluxation, cerebral atrophy, subependymal nodular high-intensity lesions, cerebellar partial agenesis, colpocephaly, megacysterna magna, hypoplasia of *corpus callosum*, frontal cortical dysplasia, dysarthria, myoclonus, very high pain threshold, placenta previa, oligohydramnios, rigid behavior, anxiety, apnea, urticaria, clubbing nails, pigmented nevi, lipomyelomeningocele, lordosis, cervical ribs.

## 4. Discussion

SHMS or *PACS1* Neurodevelopmental disorder was first reported in 2012 and it is a rare cause of ID. Likely, many patients still remain unreported and, although more than 150 patients are currently known, only 61 patients have been published up to date [1,2,3,4,5,6,7,9,10,11,12,14,16]. The disease causing *PACS1* variant always occurred de novo and therefore was excluded in the unaffected analyzed parents.

In this paper we review the clinical and molecular aspects of seven more patients with SHMS as well as all the previously reported individuals, and provide a summary of clinical findings (Table 1). The findings observed in a small number of patients are also listed in Table 1. Patients with SHMS may be clinically diagnosed when ID and speech anomalies are associated with a recognizable facial phenotype consistent in round face, full arched eyebrows, ocular hypertelorism, downslanting palpebral fissures, eye anomalies, bulbous nasal tip, wide mouth with long and flat philtrum and tented and thin upper lip with abnormal vermillion.

The PACS1 protein is a trans-Golgi-membrane traffic regulator that directs protein cargo and several viral envelope proteins. It is upregulated during human embryonic brain development and has low expression after birth. Patients with SHMS produce a protein with dominant-negative or gain-of-function effects that lead to the clinical findings characteristic of the syndrome [2]. Currently, there are ongoing efforts to develop an anti-sense therapy that would work by binding to the mutant RNA, and stopping the production of the toxic protein that causes the symptoms of the disease (https://www.pacs1foundation.org/research).

In summary, here we report on seven novel patients with SHMS with pathogenic variant in *PACS1* and review clinical and molecular findings of all the previously reported patients, providing a comprehensive review of the syndrome. In order to help physicians to recognize the syndrome, the clinical features of the 61 patients are grouped in very frequent (≥75% of patients), frequent (50–74%), infrequent (26–49%) and rare (less than ≤25%).

## Figures and Tables

**Table 1:** Summary of the clinical features of 61 patients (37 males and 24 females) with SHMS/*PACS1* Neurodevelopmental disorder included in this paper. Clinical features are organized according to the HPO nomenclature and grouped in very frequent (≥75% of patients), frequent (50–74%), infrequent (26–49%) and rare (less than ≤25%) findings. In total, 58 patients were diagnosed after birth and three prenatally.

Trait (HPO)	N	%
Very Frequent (≥75%)		
Intellectual disability (HP:0001249)	56/58	97
Dysmorphic facial features (HP:0001999)	49/60	82
Speech delay (HP:0000750)	42/55	76
**Frequent (50–74%)**		
Seizures (HP:0001250)	33/58	57
**Infrequent (26–49%)**		
Global development delay (HP:0001263)	26/58	45
Cognitive impairment (HP:0100543)	22/58	38
Hypotonia (HP:0001290)	22/58	38
Motor delay (HP:0001270)	21/57	37
Cryptorchidism (HP:0000028)	12/39	30
Amenorrhea (HP:0000141)	3/10	30
Constipation (HP:0002019)	15/57	26
Structural brain anomalies (HP:0012443)	13/49	26
**Rare (≤25%)**		
Downslanted palpebral fissures (HP:0000494)	14/61	23
Oral aversion (HP:0012523)	12/54	22
Autistic Spectrum Disorder (HP:0000729)	12/56	21
Bulbous nose (HP:0000414)	13/61	21
Microcephaly (HP:0000252)	13/61	21
Ocular Hypertelorism (HP:0000316)	12/61	20
Eye anomalies (other than colobomata) (HP:0000478)	12/61	20
Temper tantrums-aggressions (HP:0025160)	11/55	20
Clinodactyly (HP:0030084)	12/61	20
Abnormal skull shape (HP:0002648)	11/61	18
Single transverse palmar crease (HP:0000954)	11/61	18
Thin upper lip (HP:0000219)	11/61	18
Gastroesophageal reflux (HP:0002020)	09/55	16
Low-set ears (HP:0000369)	9/61	15
Arched eyebrows (HP:0002553)	9/61	15
Failure to thrive (HP:0001508)	8/57	14
*Pes planus* (HP:0001763)	8/57	14
Myopia (HP:0000545)	7/49	14
Cerebellar hypoplasia (HP:0001321)	6/43	14
Congenital heart defect (HP:0001627)	7/54	13
Wide mouth (HP:0000154)	8/61	13
Retinal coloboma (HP:0000480)	6/47	13
Full eyebrows (HP:0004523)	7/61	12
Long eyelashes (HP:0000527)	7/61	12
Umbilical hernia- Inguinal hernia (HP:0001537)	7/61	12
Short stature (HP:0004322)	7/57	12
Hypoplastic *labia minora* (HP:0000064)	3/25	12
Atrial septal defect (HP:0001631)	6/53	11
Patent *ductus arteriosus* (HP:0001643)	6/53	11
Ventricular septal defect (HP:0001629)	6/53	11
Low birth weight (HP:0001518)	6/56	11
Diastema (HP:00006999	6/55	11
Downturned corners of the mouth (HP:0002714)	7/61	11
Anteverted nares (HP:0000463)	7/61	11
Coloboma of choroid (HP:0000567)	5/49	10
Epicanthus (HP:0000286)	6/61	10
Broad nasal bridge (HP:0012811)	6/61	10
Coloboma of optic nerve (HP:0000588)	5/49	10
Upturned nose (HP:0000463)	6/61	10
Flat *philtrum* (HP:0000319)	6/61	10
Recurrent infections (HP:0002719)	6/61	10
Iris coloboma (HP:0000612)	5/57	9
Upswept anterior hairline (HP:0002236)	5/61	8
Tented mouth (HP:0010804)	5/61	8
G-tube feeding (HP:0040288)	5/61	8
*Pectus excavatum* (HP:0000767)	5/61	8
Synophrys (HP:0000664)	4/61	7
Camptodactyly (HP:0012385)	4/61	7
Short neck (HP:0000470)	4/61	7
Clumsiness (HP:0002312)	4/61	7
Absent speech (HP:0001344)	4/56	7
Behavioral abnormality (HP:0000708)	4/56	7
Sleep disturbance (HP:0002360)	4/56	7
Abnormality of the kidney (HP:0000077)	4/59	7
Scoliosis (HP:0002650)	4/54	7
Widely spaced nipples (HP:0006610)	4/61	7
Abnormality of the cerebral white matter (HP:0002500)	3/43	7
Slender finger (HP:0001238)	4/58	7
Coarctation of aorta (HP:0001680)	3/53	6
Posteriorly rotated ears (HP:0000358)	4/61	6
Flat occiput (HP:0005469)	4/61	6
Eversion of lateral third of lower eyelids (HP:0007655)	3/61	5
Nystagmus (HP:0000639)	3/61	5
Strabismus (HP:0000486)	3/61	5
Round face (HP:0000311)	3/61	5
Low anterior hairline (HP:0000294)	3/61	5
Micrognathia (HP:0000347)	3/61	5
Large for gestational age (HP:0001520)	3/61	5
Tapered finger (HP:0001182)	3/57	5
Dystonia (HP:0001332)	3/54	5
Involuntary movements (HP:0004305)	3/57	5
Hydrocephalus (HP:0000238)	2/42	5
Ataxia (HP:0001251)	2/43	5
Bicuspid aortic valve (HP:0001647)	2/53	4
Patent *foramen ovale* (HP:0001655)	2/53	4
Astigmatism (HP:0000483)	2/49	4
Microcornea (HP:0000482)	2/49	4
Falls (HP:0002527)	2/49	4
Increased nuchal translucency (HP:0010880)	2/48	4
Microphthalmia (HP:0000568)	2/57	3
Upslanted palpebral fissures (HP:0000582)	2/61	3
Triangular face (HP:0000325)	2/61	3
Widow’s peak (HP:0000349)	2/61	3
Cleft lip (HP:0410030)	2/61	3
Concave nasal ridge (HP:0011120)	2/61	3
Long *philtrum* (HP:0000343)	2/61	3
Short *philtrum* (HP:0000322)	2/61	3
Macrocephaly (HP:0000256)	2/61	3
Tall stature (HP:0000098)	2/61	3
Recurrent otitis media (HP:0000403)	2/61	3
Brachydactyly (HP:0001156)	2/61	3
Broad *hallux* (HP:0010055)	2/61	3
Finger joint hypermobility (HP:0006094)	2/61	3
Long foot (HP:0001833)	2/61	3
Large hands (HP:0001176)	2/61	3
Overlapping toes (HP:0001845)	2/61	3
Epileptic encephalopathy (HP:0200134)	2/61	3
Placental bleeding (HP:0025328)	2/61	3
Inappropriate laughter (HP:0000748)	3/59	3
Repetitive compulsive behavior (HP:0008762)	2/58	3
Self-injurious behavior (HP:0100716)	2/58	3
Pulmonary hypoplasia (HP:0002089)	2/58	3
Short ears (HP:0400004)	2/61	3
Almond-shaped eyes (HP:0007874)	2/61	3
Telecanthus (HP:0000506)	2/61	3
Ptosis *palpebralis* (HP:0000508)	2/61	3
Pleural effusion (HP:0002202)	2/61	3

## Data Availability

The data presented in this study are available on request from the corresponding author. The data are not publicly available due to patient’s inform consent restrictions.

## Supplementary Materials

The following are available online at https://www.mdpi.com/article/10.3390/genes12050738/s1, title, Table S1: Clinical characteristics of the seven novel patients with SHMS reported in this paper, Table S2: The ENOD Consortium members, S3: The SIDE (Spanish Intellectual Disability Exome) Consortium members.
