# Supplementary material for: Schuurs–Hoeijmakers Syndrome (PACS1 Neurodevelopmental Disorder): Seven Novel Patients and a Review
Source: Genes (Basel). 2021 May 13;12(5):738. doi: 10.3390/genes12050738 (PMC8153584; doi:10.3390/genes12050738)
Supplement: Supplementary file 1 [file genes-12-00738-s001.zip › genes-1195929-Supplementary Materials .pdf]

Supplementary Materials Table S1. Clinical characteristics of the seven novel patients with SHMS reported in this paper.

System	Subsystem	Trait	Patient 1	Patient 2	Patient 3	Patient 4	Patient 5	Patient 6	Patient 7
		Male	+	+				+	
		Female			+	+	+		+
		Pathogenic variant	c.607C>T	c.607C > T	c.607C > T	c.607C > T	c.607C > T	c.607C > T	c.607C > T
Craniofacial	Ear	Low-set ears	+	+	+	-	+	-	+
Craniofacial	Ear	Posteriorly rotated ears	-	+	+	-	+	-	+
Craniofacial	Ear	Almond eyes	-	-	-	-	-	-	+
Craniofacial	Eyes	Arched eyebrows	-	-	+	-	-	-	+
Craniofacial	Eyes	Coloboma	+	-	+	+	+	-	-
Craniofacial	Eyes	Coloboma of choroid	+	-	+	+	+	-	-
Craniofacial	Eyes	Coloboma of optic nerve	-	NE	+	+	-	-	-
Craniofacial	Eyes	Retinal coloboma	+	-	+	+	+	-	-
Craniofacial	Eyes	Downslanting palpebral fissures	+	-	+	+	+	-	+
Craniofacial	Eyes	Ectropion	-	-	-	-	-	+	-
Craniofacial	Eyes	Epicanthus	+	+	-	-	+	-	+
Craniofacial	Eyes	Eversion of the lateral third of lower eyelids	-	-	+	-	-	+	-
Craniofacial	Eyes	Eye anomalies (other than colobomata)	+	+	-	-	-	-	-
Craniofacial	Eyes	Full eyebrows	+	+	+	-	+	-	-
Craniofacial	Eyes	Highly arched eyebrow	+	-	+	-	-	+	-
Craniofacial	Eyes	Lens subluxation	+	-	-	-	-	-	-
Craniofacial	Eyes	Peters' anomaly	-	+	-	-	-	-	-
Craniofacial	Eyes	Ocular Hypertelorism	+	-	+	-	+	-	+
Craniofacial	Eyes	Iris coloboma	+	-	+	-	-	-	-
Craniofacial	Eyes	Long eyelashes	+	-	+	-	+	-	-
Craniofacial	Eyes	Microcornea	**?**	+	+	-	-	-	-
Craniofacial	Eyes	Microphthalmia	+	**NE**	-	+	-	-	-
Craniofacial	Eyes	Myopia	**NE**	**NE**	+	-	-	+	-
Craniofacial	Eyes	Nystagmus	+	**NE**	-	-	-	-	-
Craniofacial	Eyes	Strabismus	**NE**	**NE**	+	-	-	-	-
Craniofacial	Eyes	Synophrys	+	-	+	-	-	+	-
Craniofacial	Eyes	Ptosis palpebralis	-	-	-	-	-	-	+
Craniofacial	Eyes	Telecanthus	+	-	-	-	-	-	+
Craniofacial	Eyes	Upslanting palpebral fissures	-	-	+	-	-	-	-
Craniofacial	face	Dysmorphic facial features	+	+	+	+	+	+	+
Craniofacial	face	Round face	+	-	-	+	-	-	-
Craniofacial	face	Triangular face	-	+	-	-	-	-	-
Craniofacial	Hair	Low anterior hairline	-	-	+	-	+	-	-
Craniofacial	Hair	Upswept anterior hairline	+	+	+	-	+	-	-
Craniofacial	Hair	Widow's peak	-	-	-	-	+	-	-
Craniofacial	Lip	Thin upper lip	-	-	+	+	+	-	+
Craniofacial	Mandible	Retrognathia	-	-	-	+	-	-	-
Craniofacial	Mandible	Micrognathia	+	-	-	+	-	-	-
Craniofacial	Mouth	Downturned corners of the mouth	-	-	+	-	-	-	+
Craniofacial	Mouth	Tented mouth	-	-	-	-	+	-	+
Craniofacial	Mouth	Wide mouth	+	-	+	-	+	-	-
Craniofacial	Nose	Anteverted nares	+	-	+	+	-	+	+
Craniofacial	Nose	Broad nasal bridge	+	-	+	-	+	-	+
Craniofacial	Nose	Bulbous nose	+	-	+	+	+	-	+
Craniofacial	Nose	Saddle nose	**?**	-	+	-	-	-	-
Craniofacial	Nose	Upturned nose	+	-	+	+	-	-	+
Craniofacial	*Philtrum*	Flat *philtrum*	+	-	+	-	+	+	+
Craniofacial	*Philtrum*	Long *philtrum*	-	-	+	-	+	-	-
Craniofacial	*Philtrum*	Short *philtrum*	+	+	-	-	-	-	-
Craniofacial	Skull	Abnormal skull shape	+	-	+	-	+	-	-
Craniofacial	Skull	Flat occiput	+	**NE**	+	-	-	-	+
Craniofacial	Skull	Macrocephaly	-	+	-	-	-	-	-

Craniofacial	Skull	Microcephaly	-	-	+	-	-	-	+
Craniofacial	Teeth	Diastema	**NE**	**NE**	+	-	+	+	+
Craniofacial	Teeth	Misplaced teeth	-	+	-	-	-	-	-
Genitalia		Amenorrhea	**NE**	**NE**	-	-	+	-	**NA**
Genitalia		Cryptorchidism	-	+	-	-	-	+	**NA**
Genitalia		Hypoplastic labia minora	**NE**	**NE**	+	-	-	-	-
Genitalia		Septate uterus	-	-	+	-	-	-	**NA**
GI		Constipation	**NE**	+	-	-	-	+	-
GI		Umbilical hernia-inguinal hernia	-	+	-	-	-	-	-
Growth		Failure to thrive	-	+	+	-	-	-	-
Growth		Poor feeding	-	-	-	-	-	-	-
Growth		Short stature	-	+	+	-	+	-	-
Limbs		Brachydactyly	+	-	-	-	+	-	-
Limbs		Camptodactyly	-	-	+	-	-	-	-
Limbs		Clinodactyly	+	-	-	-	+	-	-
Limbs		Hypermobility of finger joints	+	**NE**	**NE**	-	-	-	-
Limbs		Large feet	**NE**	-	+	-	-	-	-
Limbs		Large hands	-	-	+	-	-	-	-
Limbs		Overlapping toes	-	-	-	-	+	-	-
Limbs		*Pes planus*	**NE**	+	-	-	-	-	-
Limbs		Single transverse palmar crease	+	-	-	-	+	+	-
Limbs		Slender finger	+	+	-	-	-	+	+
Limbs		Fibular subluxation	-	-	+	-	-	-	-
Neck		Short neck	+	-	-	+	-	-	-
Neuro		Structural brain anomalies	**NE**	-	-	-	+	-	-
Neuro		Clumsiness	**NE**	+	+	-	+	-	-
Neuro		Cognitive impairment	+	+	+	+	+	+	-
Neuro		Dysarthria	**NE**	**NE**	-	-	-	+	-
Neuro		Dystonic movements	+	-	-	-	-	-	-
Neuro		Epileptic encephalopathy	-	-	+	-	-	-	-
Neuro		Falls	**NE**	**NE**	+	-	-	-	-
Neuro		Global development delay	+	+	+	+	+	+	-
Neuro		Hypotonia	+	-	+	-	+	+	-
Neuro		Intellectual disability	+	+	+	+	+	+	+
Neuro		Involuntary movements	+	-	+	-	-	-	-
Neuro		Motor delay	+	+	+	+	+	+	+
Neuro		Seizures	+		+	-	-	+	-
Neuro		Speech delay	**NE**	+	+	+	+	+	+
Psychiatric		Abnormal behavior	-	-	-	+	-	-	-
Psychiatric		Attention deficit hyperactivity disorder	**NE**	-	-	-	-	-	-
Psychiatric		Autism	**NE**	+	**NE**	-	-	-	-
Psychiatric		Laughing episodes	**NE**	-	+	-	-	-	+
Psychiatric		Repetitive behavior	**NE**	**NE**	-	+	-	-	-
Psychiatric		Self-injurious behavior	**NE**	**NE**	-	+	-	-	-
Psychiatric		Temper tantrums-aggression	**NE**	**NE**	-	+	-	-	-
Spine and thorax		Scoliosis	**NE**	-	+	-	-	-	-

(+): present; **(-)**: not present; **NE:** not evaluated/not reported.

Supplementary Table S2. The ENOD Consortium.

**Enrique Herreras**	enrique@ciberer.es	CIBERER, Centro de Investigación Biomédica en Red de Enfermedades Raras, ISCIII, 28029 Madrid, Spain	
Joaquín Dopazo	joaquin.dopazo@juntadeandalucia.es	CIBERER, Centro de Investigación Biomédica en Red de Enfermedades Raras, ISCIII, 28029 Madrid, Spain	Clinical Bioinformatics Area, Fundacion Progreso y Salud, 41013 Sevilla, Spain
Javier Perez-Florido	javier.perez.florido.sspa@juntadeandalucia.es	CIBERER, Centro de Investigación Biomédica en Red de Enfermedades Raras, ISCIII, 28029 Madrid, Spain	Clinical Bioinformatics Area, Fundacion Progreso y Salud, 41013 Sevilla, Spain
Rosario Carmona	rosariom.carmona@juntadeandalucia.es	CIBERER, Centro de Investigación Biomédica en Red de Enfermedades Raras, ISCIII, 28029 Madrid, Spain	Clinical Bioinformatics Area, Fundacion Progreso y Salud, 41013 Sevilla, Spain

Virginia Aquino	virginia.aquino@juntadeandalucia.es	CIBERER, Centro de Investigación Biomédica en Red de Enfermedades Raras, ISCIII, 28029 Madrid, Spain	Clinical Bioinformatics Area, Fundacion Progreso y Salud, 41013 Sevilla, Spain
Francisco Ortuño	franciscom.ortuno@juntadeandalucia.es	CIBERER, Centro de Investigación Biomédica en Red de Enfermedades Raras, ISCIII, 28029 Madrid, Spain	Clinical Bioinformatics Area, Fundacion Progreso y Salud, 41013 Sevilla, Spain
Daniel Lopez-Lopez	daniel.lopez.lopez@juntadeandalucia.es	CIBERER, Centro de Investigación Biomédica en Red de Enfermedades Raras, ISCIII, 28029 Madrid, Spain	Clinical Bioinformatics Area, Fundacion Progreso y Salud, 41013 Sevilla, Spain
Gerrit Bostelmann	gerrit.bostelmann@juntadeandalucia.	CIBERER, Centro de Investigación Biomédica en Red de Enfermedades Raras, ISCIII, 28029 Madrid, Spain	Clinical Bioinformatics Area, Fundacion Progreso y Salud, 41013 Sevilla, Spain

Supplementary Table S3. The SIDE (Spanish Intellectual Disability Exome) Consortium.

Hospital/Institution	Name and Surname	E-mail
Hospital Universitario de Badajoz	Enrique Galán Gómez	enrique.galangomez@gmail.com
Hospital Universitario de Badajoz	María Pilar Méndez Pérez	mariapilar.mendez@salud-juntaex.es
Hospital Universitario Fundacion Jimenez Diaz	Berta Almoguera	BAlmoguera@quironsalud.es
Hospital Universitario Fundacion Jimenez Diaz	Carmen Ayuso	cayuso@fjd.es
Hospital Universitario Fundacion Jimenez Diaz	Isabel Lorda-Sanchez	ilorda@fjd.es
Hospital Universitario Fundacion Jimenez Diaz	Fiona Blanco-Kelly	FBlancoK@quironsalud.es
Hospital Universitario Fundacion Jimenez Diaz	Saoud Swafiri	stahsin@quironsalud.es
Hospital Universitario Fundacion Jimenez Diaz	Marta Corton	mcorton@quironsalud.es
Hospital Universitario Fundacion Jimenez Diaz	Mª Jose Trujillo-Tiebas	MJTrujillo@fjd.es
Hospital Universitario Fundacion Jimenez Diaz	Elvira Rodriguez-Pinilla	
Hospital Universitario Fundacion Jimenez Diaz	Berta Almoguera	BAlmoguera@quironsalud.es
Hospital Universitario Fundacion Jimenez Diaz	Carmen Ayuso	cayuso@fjd.es
Hospital Universitario Fundacion Jimenez Diaz	Isabel Lorda-Sanchez	ilorda@fjd.es
Hospital Universitario Fundacion Jimenez Diaz	Fiona Blanco-Kelly	FBlancoK@quironsalud.es
Hospital Universitario Fundacion Jimenez Diaz	Saoud Swafiri	stahsin@quironsalud.es
Hospital Universitario Fundacion Jimenez Diaz	Marta Corton	mcorton@quironsalud.es
Hospital Universitario Fundacion Jimenez Diaz	Mª Jose Trujillo-Tiebas	MJTrujillo@fjd.es
Hospital Universitario Fundacion Jimenez Diaz	Fermina Lopez Grondona	fermina.lopez@quironsalud.es
Hospital Universitario Fundacion Jimenez Diaz	Elvira Rodriguez-Pinilla	
Hospital San Pedro de Alcántara	Ignacio Arroyo Carrera	ignacio.arroyo@salud-juntaex.es
Hospital de Mérida	MIGUEL FERNÁNDEZ-BURRIEL TERCERO	MIGUEL.FERNANDEZB@SALUD-JUNTAEX.ES
Hospital de Mérida	FRANCISCO ESPEJO LÓPEZ	FRANCISCO.ESPEJO@SALUD-JUNTAEX.ES
Hospital Universitario Ramón y Cajal	Miguel Angel Moreno Pelayo	mmorenop@salud.madrid.org
Hospital Universitario Ramón y Cajal	Matías Morín Rodríguez	matias.morin@salud.madrid.org
Hospital Universitario Ramón y Cajal	Dolores Rey Zamora	dolores.rey@salud.madrid.org
Hospital Universitario Ramón y Cajal	María Lachgar Ruiz	maria.lachgar@salud.madrid.org
Hospital Universitario 12 de Octubre	Moreno García, Marta	m.moreno@salud.madrid.org
Hospital Universitario 12 de Octubre	Quesada Espinosa, Juan Francisco	juanf.quesada@salud.madrid.org
Hospital Universitario 12 de Octubre	Ana Rosa Arteche	anarosa.arteche@salud.madrid.org
Hospital Universitario 12 de Octubre	Maria Isabel Álvarez Mora	MIALVAREZ@clinic.cat
Hospital Universitario 12 de Octubre	José Miguel Lezana Rosales	josemiguel.lezana@salud.madrid.org
Hospital Universitario 12 de Octubre	Carmen Palma Milla	carmen.palma@salud.madrid.org
Hospital Universitario 12 de Octubre	M Teresa Sánchez Calvín	mscalvin@salud.madrid.org
Hospital Universitario 12 de Octubre	Irene Gómez Manjón	igomez@salud.madrid.org
Hospital Universitario 12 de Octubre	Jaime Sánchez del Pozo	jaime.sanchez@salud.madrid.org
Hospital Universitario 12 de Octubre	Jaime Cruz Rojo	jaime.cruz@salud.madrid.org
Hospital Universitario Principe de Asturias	Blanca García García	bgarciagarcia@salud.madrid.org
Hospital Universitario Principe de Asturias	Luis Antonio Varela Sanz	luisantonio.varela@salud.madrid.org
Hospital Universitario de Móstoles	Mª Teresa Darnaude Ortiz	mariateresa.darnaude@salud.madrid.org

Hospital Universitario de Móstoles	Aranzazu Díaz de Bustamante	adiazb@salud.madrid.org
Hospital Universitario de Móstoles	Rebeca Villares Alonso	rebeca.villares@salud.madrid.org
Hospital Universitario de Móstoles	Julian Torres Mohedas	julian.torres@salud.madrid.org
Hospital Clínico San Carlos	María Carmen Cotarelo Pérez	mariacarmen.cotarelo@salud.madrid.org
Hospital Clínico San Carlos	María del Mar Fenollar Cortés	mariadelmar.fenollar@salud.madrid.org
Hospital Clínico San Carlos	Clara Herrero Forte	clara.herrero@salud.madrid.org
Hospital Clínico San Carlos	Raluca Oancea Ionescu	raluca.oancea@salud.madrid.org
Hospital Universitario de Getafe	Belén Gil-Fournier	belen.gilfournier@salud.madrid.org
Hospital Universitario de Getafe	Soraya Ramiro León	soraya.ramiroleon@salud.madrid.org
Hospital Universitario de Getafe	Beatriz Martínez Menéndez	bmmenendez@salud.madrid.org
Hospital Universitario de Getafe	Francisco Javier Martínez Sarries	fmsarries@salud.madrid.org
Hospital Universitario La Paz	Maria Palomares-Bralo	mpalomares.ingemm@gmail.com
Hospital Universitario La Paz	Marta Pacio-Míguez	martapaciomiguez@gmail.com
Hospital Universitario La Paz	Sixto García-Miñaúr	sixto.garciamin@gmail.com
Hospital Universitario La Paz	Fernando Santos-Simarro	fernando.santos@salud.madrid.org
Hospital Universitario La Paz	Angela del Pozo	ingemm.adelpozo@gmail.com
Hospital Universitario La Paz	Mario Solís	ingemm.msolis@gmail.com
Hospital Universitario La Paz	Julián Nevado Blanco	jnevadobl@gmail.com
Hospital Universitario La Paz	Pablo Lapunzina	plapunzina@gmail.com
Hospital Universitario La Paz	Jair Tenorio	jaira.tenorio@salud.madrid.org
Hospital Universitario Gregorio Marañón	Verónica Seidel	veronicaadriana.seidel@salud.madrid.org
Hospital Universitario Gregorio Marañón	María Orera	maria.orera@salud.madrid.org
Hospital Universitario Infanta Sofia	Cristina González	cgonzalez@brsalud.es
Hospital Universitario La Princesa	Eva Arranz Muñóz	eva.arranz@salud.madrid.org
Hospital Universitario La Princesa	Maria Ángeles Sanz de Benito	msanzbenito@salud.madrid.org
